# Accuracy of Navigated High-Speed Drill-Assisted Cervical and Upper Thoracic Pedicle Screw Placement—A Single Center Experience with 1112 Pedicle Screws

**DOI:** 10.3390/jcm14186597

**Published:** 2025-09-19

**Authors:** Stefan Aspalter, Nico Stroh-Holly, Katja Höllmüller, Armin Davachi, Philip Rauch, Stephan Heisinger, Andreas Gruber, Wolfgang Senker

**Affiliations:** 1 Department of Neurosurgery, Kepler University Hospital GmbH, Johannes Kepler University, 4040 Linz, Austria; stefan.aspalter@kepleruniklinikum.at (S.A.); k.hoellmueller01@gmail.com (K.H.); armin.davachi@kepleruniklinikum.at (A.D.); philip-rudolf.rauch@kepleruniklinikum.at (P.R.); andreas.gruber_1@kepleruniklinikum.at (A.G.); wolfgang.senker@kepleruniklinikum.at (W.S.); 2 Department of Orthopedics and Trauma Surgery, Medical University of Vienna, 1090 Vienna, Austria; stephan.heisinger@meduniwien.ac.at

**Keywords:** cervical pedicle screws, navigation, O-arm navigation, navigated high-speed drill, screw accuracy, Gertzbein–Robbins classification, workflow, cervical spine

## Abstract

**Background/Objectives**: While biomechanically superior, cervical pedicle screw placement is technically challenging, and therefore typically performed only in centers with the aid of navigation. The purpose of this study was to analyze the accuracy and safety of navigated cervical pedicle screw (CPS) placement using intraoperative imaging with a workflow using a navigated high-speed drill in a large single-center cohort. **Methods**: We conducted a retrospective analysis of 205 patients undergoing posterior cervical or cervicothoracic instrumentation between January 2018 and June 2024. Accuracy was assessed using the Gertzbein–Robbins classification, with grades 0 and 1 considered satisfactory. Surgical workflow, intraoperative imaging, and complications were analyzed. **Results**: A total of 1112 pedicle screws, including 888 cervical and 224 upper thoracic screws, were evaluated. 801 were grade 0 (72.0%), 250 grade 1 (22.5%), 56 grade 2 (5.0%), and 5 grade 3 (0.4%). Cervical screws achieved satisfactory placement grades 0 and 1 in 93.1%, and upper thoracic screws in 100% (92.0% grade 0, 8.0% grade 1). Grade 3 breaches occurred in C2, C3, C5, C6, and C7, with one case each. There were no cases of implant-related neurovascular injuries. **Conclusions**: This study demonstrates high screw accuracy with a low observed complication rate. No revision surgeries were required due to screw malposition, but 7 cases of screw loosening occurred. However, the retrospective design and reliance on intraoperative imaging limit the generalizability of the findings.

## 1. Introduction

Pedicle screw placement in the cervical spine was first described by Abumi et al. in 1994 for traumatic injuries [1]. Compared to lateral mass screws, cervical pedicle screws (CPS) offer higher biomechanical stability, with lower rates of loosening and higher fatigue resistance [2,3]. However, the complex anatomy of the cervical spine—characterized by small pedicle diameters, variable angulations, and proximity to neurovascular structures—makes CPS placement technically demanding and associated with increased risk of vertebral artery injury and neural compromise [4,5].

A meta-analysis by Soliman et al., involving more than 16,000 screws, reported a vertebral artery injury rate of up to 0.4% for CPS placement, while lateral mass screws show no such complications [6]. The introduction of spinal navigation systems has significantly improved the safety and precision of CPS placement and has facilitated increased utilization of cervical pedicle screws [6]. Navigation-assisted techniques have demonstrated improved accuracy and reduced complication rates compared to traditional fluoroscopy-guided methods. Kotani et al. reported a pedicle perforation rate of 1.2% with computer-assisted navigation versus 6.7% with manual techniques, and Tarawneh et al. found neurovascular events in 0.3% of navigated cases compared to 1.9% with fluoroscopy [7,8]. Recent technological advances have introduced robot-assisted systems and 3D-printed templates, which further enhance placement accuracy [9].

Despite these advancements, CPS placement remains a technically demanding procedure due to the risks mentioned earlier.

Few studies have reported on the radiographic accuracy of CPS placement using detailed classification systems, such as the Gertzbein–Robbins classification [10,11,12,13]. For example, Bredow et al. [11] analyzed 207 screws placed from C1 to C7 and reported an overall accuracy (grade 2 or better) of 78.51% for screws at C3–C7 and 93.9% for C1/C2 screws.

Moreover, the role of a navigated high-speed drill, which potentially minimizes vertebral displacement during the implantation process to preserve accuracy of the navigation system—has not been extensively studied. The present study aims to assess the accuracy and safety of a navigated high-speed drill-assisted workflow for CPS placement in a large single-center cohort. By analyzing over 1100 screws placed in the cervical and upper thoracic spine, we seek to validate previous findings and provide additional insight into rare complications that may be underrepresented in smaller series.

## 2. Materials and Methods

This study adhered to all relevant national regulations, institutional policies, and the tenets of the Helsinki Declaration. The study was designed as a single-center retrospective analysis. Included were patients who underwent posterior cervical or cervicothoracic instrumentation with pedicle screws at our institution between January 2018 and June 2024. Exclusion criteria were patient age < 18 and posterior cervical or cervicothoracic fusion procedures in which no pedicle screws were placed.

**Figure 1:**
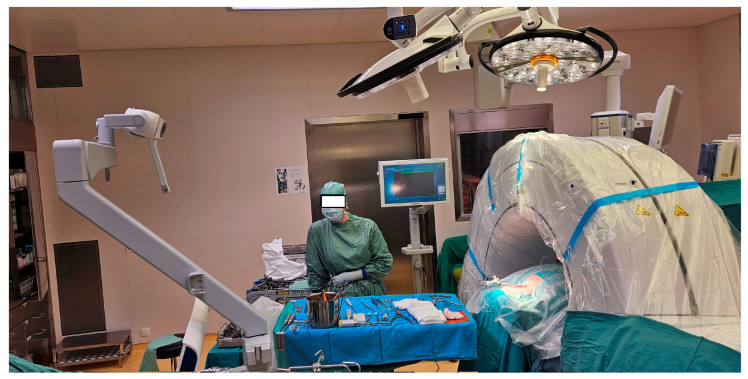
Typical setup in the operating room. The intraoperative imaging system (O-Arm) is sterile draped and in the parking position, placed caudally. The screen of the navigation unit is placed contralateral to the surgeon. The scrub nurse, instruments, and implant trays are located cranially.

**Figure 2:**
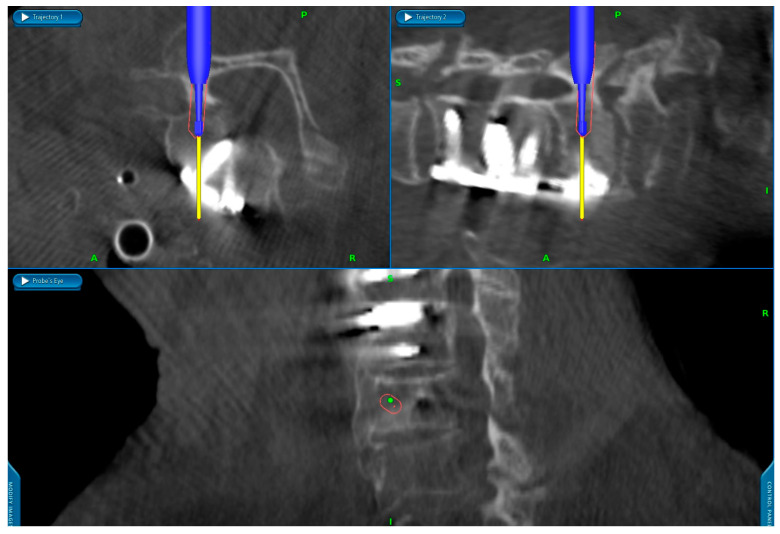
Screenshot of the navigation screen, showing the drilling for a C6 pedicle screw on the left side using a navigated high-speed drill. The trajectory is visualized in axial and sagittal views, as well as in the Probe’s Eye view. In this specific case, a junctional fracture at C6/7 occurred following an anterior fusion from C4 to C6, necessitating posterior fusion. A = anterior, R = right, P = posterior, S = superior, I = inferior.

**Figure 3:**
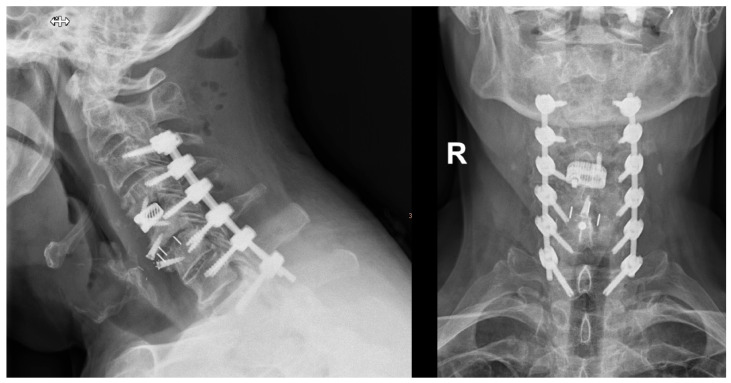
Postoperative X-ray after implantation of pedicle screws from C3-T1. In this specific case, spondylitis several years after anterior cervical discectomy and fusion with implant loosening of the upper cage at C4/5 made revision surgery necessary with implantation of a new cage at C4/5, and subsequent posterior instrumentation. R = right.

**Surgical Technique:** After the patient’s head was fixed in a carbon Mayfield clamp, all patients were positioned prone in a reverse-Trendelenburg position on a carbon operating table. The operative field was disinfected and draped. An intraoperative cone beam CT (O-arm^®^; Medtronic, Louisville, KY, USA) was positioned within the operative field, sterilely draped, and parked caudally to minimize interference with the surgical team. Figure 1 shows the typical patient positioning and setup of the operating room. After a midline skin incision, the neck muscles overlying the spine segments were dissected to expose the posterior cervical spine. A spinous process clamp with a navigation array was then attached. For most cases, either the spinous process of C2 or C7 were used, as these typically are big enough to allow in most cases sufficient bone purchase of the spinous process clamp Typically, in procedures where only the upper cervical spine was instrumented, the reference clamp was positioned on C2, and in cases in which also the middle or lower cervical spine was instrumented, the clamp was positioned on C7. A 3D scan was then performed, and the screw trajectories were planned using the StealthStation™ S7 navigation system (Medtronic, Minneapolis, MN, USA). A high-speed navigated drill (Stealth-Midas™; Medtronic) was used to burr the planned trajectories (see Figure 2), followed by assessment of the pedicle cortex with a probe. Screws were inserted directly after burring the according screw hole. Screw placement typically started with the most cranial vertebra on the right side, followed sequentially by placement of the other screws in a clockwise sequence. After all screws were implanted, another 3D scan was performed to verify screw position. If repositioning was required, screws were adjusted and rescanned. If necessary, decompression via laminectomy was performed after insertion of the screws. Rods were fixed to the screw heads once satisfactory placement of all screws was achieved. Afterwards, an intraoperative x-ray was taken to ensure correct positioning of the screws and rods. Before wound closure, either one or two Redon drains were inserted. Finally, the surgical wound was closed in a multi-layer fashion. Postoperatively, patients were mobilized on the day after surgery, and patients received routinely a soft cervical collar for 4 weeks postoperative. Figure 3 shows an example of pedicle screw instrumentation C3-T1.

**Data Collection:** We assessed patient demographics, indications for surgery, intraoperative and perioperative complications, blood loss, operation time, type, and number of revision surgeries. Furthermore, we recorded the possible replacement of screws and the number of intraoperative O-arm scans.

The radiographic accuracy of pedicle screw placement was assessed using intraoperative O-arm images. The Gertzbein–Robbins classification system [10] was employed to evaluate screw positions, with grades 0–3. Grade 0 represents no cortical violation, grade 1 a minor cortical breach up to 2 mm, grade 2 a cortical breach greater than 2 mm, and grade 3 more than 4 mm cortical breaches. Measurements for the definite screw position were taken on the last O-arm scan. Accuracy of the screw positioning was assessed using the radiological program DeepUnity Diagnost (DH Healthcare GmbH, Bonn, Germany). Each screw was checked on possible violations of the cortex on three planes (axial, sagittal, coronal). If there was any violation of the pedicle cortex, the maximum distance between cortex and screw was measured in mm and accordingly classified using the Gertzbein Robbins Classification. In cases where it was not possible to distinguish between pedicle cortex and the screw itself due to artifacts to distinguish between grade 0 and grade 1, the worse grade (grade 1) was assigned.

## 3. Results

A total of 205 patients (75 female, 130 male) with a mean age of 67.0 years were included in the study. The most frequent indications for surgery were traumatic injuries, including fractures or discoligamentous instability, necessitating instrumentation in 92 cases (44.8%), followed by degenerative disorders in 66 cases (32.1%). Other indications included tumor-related instability (n = 20, 9.8%), infections (n = 9, 4.4%), pseudarthrosis (n = 8, 3.9%), mechanical failure of prior instrumentation without pedicle screws (n = 7, 3.4%), and instability from rheumatic disorders (n = 3, 1.5%).

The mean blood loss was 474 mL, ranging from 30 mL to 3000 mL. The mean operative time (from incision to suture) was 167.9 min. Additional decompression was performed in 99 cases (48.3%). Instrumented segment lengths ranged from 1 to 9 levels, distributed as follows: 1 level (n = 67), 2 levels (n = 36), 3 levels (n = 49), 4 levels (n = 24), 5 levels (n = 17), 6 levels (n = 8), 7 levels (n = 3), and 8 levels (n = 1).

All 205 patients received at least one cervical pedicle screw, with a range of 1 to 16 screws per patient. Including cervicothoracic fusion procedures, a total of 1112 cervical and upper thoracic pedicle screws were placed, of which 888 were cervical screws, and 224 were thoracic screws. The distribution of screws across cervical levels was relatively even, with C2 accounting for 19.4%, C3 for 14.0%, C4 for 16.0%, C5 for 16.9%, and C6 for 18.0% of all cervical screws. Among thoracic screws, 58.9% (n = 132) were placed at T1. A minimum of two intraoperative O-arm scans were performed in 148 cases (72.2%); in the case of repositioning of the screws, three scans were performed in 46 cases (22.4%), and four scans in 11 cases (5.4%).

**Figure 4:**
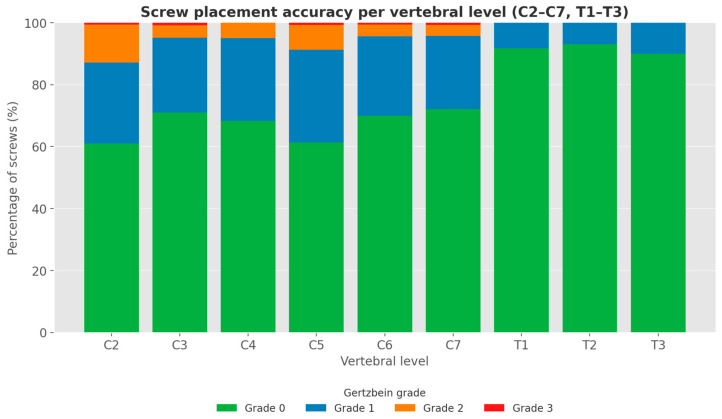
Stacked bar chart of the frequencies of the Gertzbein–Robbins-grades per vertebral level.

**Figure 5:**
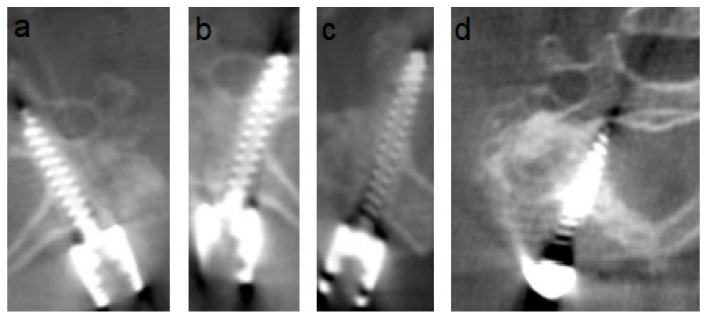
Examples of the different Gertzbein–Robbins grades on intraoperative, axial O-arm images. (**a**) shows grade 0 (no cortical breach), (**b**) grade 1 (breach < 2 mm), (**c**) grade 2 (breach 2–4 mm), and (**d**) grade 3 (breach > 4 mm).

Radiographic analysis of screw placement accuracy using the Gertzbein–Robbins classification revealed the following: Of the total 1112 screws, 801 were grade 0 (72.0%), 250 grade 1 (22.5%), 56 grade 2 (5.0%), and 5 grade 3 (0.4%). Of the 888 cervical screws, 595 were grade 0 (67.0%), 232 were grade 1 (26.1%), 56 were grade 2 (6.3%), and 5 were grade 3 (0.6%). Of 224 thoracic screws, 206 were grade 0 (92.0%), and 18 were grade 1 (8.0%). No Grade 2 or 3 breaches were observed. The detailed results are shown in Table 1 and Figure 4, and examples for each screw grade are shown in Figure 5.

The grade 3 breaches occurred at levels C2, C3, C5, C6 and C7. Two were on the right side (C6 and C7), and 3 on the left side (C2, C3 and C5).

Revision surgery was required in 18 patients (8.8%), as shown in Table 2. The most frequent cause was screw loosening, observed in 7 patients (3.4%). Of these, 5 cases (2.4%) involved cervical pedicle screws, located at C2 (n = 2), C3/C4 (n = 1), C4 (n = 1), and C5/C6 (n = 1). In the remaining 2 cases, loosening of C1 lateral mass screws following C1/2 fusion was the reason for revision. One of the revisions for screw loosening was associated with infection. Mean duration between index surgery and revision surgery due to implant loosening was 130.1 days (specific time intervals (days): 302, 163, 233, 135, 61, 152, 28, 97).

Other causes for revision included 6 cases of rebleeding (n = 6, 2.9%). All were instances of epidural hematoma. 3 occurred on the same day as index surgery, 2 on the following day, and 1 on the third day after operation. All cases presented with new, temporary sensory or motor dysfunction postoperative. All neurological deficits resolved after evacuation of the hematoma.

Wound healing disorders were seen in four cases (2.0%) which necessitated revision. In one case, prolonged therapy with a vacuum-assisted closure device was necessary for 1 month. Time between index and revision surgery were 11, 28, 21 and 35 days.

No case of neural injury, presenting either with sensory or motor deficits or radiculopathy due to mispositioned implants was found. Also, in the patients with grade 3 breaches, no secondary revision surgery due to implant malposition was performed.

Four instances (0.45% of 888 cervical screws) of intraoperative bleeding after burring of the screw hole raised suspicion of vascular injury; however, postoperative imaging (in 3 cases CT-angiography, in 1 case conventional angiography) showed in all cases unremarkable results of the vertebral arteries. Follow up was available for all 4 patients, and within the follow up period, (13 months; 26 months, 36 months, 28 months) the postoperative course was uneventful.

## 4. Discussion

This retrospective single-center study evaluated the accuracy and safety of navigation-assisted, high-speed drill-assisted cervical and upper thoracic pedicle screw placement. The above-described workflow was routinely employed at our institution, resulting in the fact that CPS are placed in nearly all posterior cervical instrumentation procedures, contributing to the high number of CPS cases included in this study. Our analysis of over 1100 pedicle screws demonstrates that this workflow achieves a high rate of accurate screw placement with 93.1% of cervical screws and 100% of upper thoracic screws classified as grade 0 or 1, resulting in a combined accuracy of 94.5%. While grade 0 represents ideal placement (no cortical breach), grade 1 breaches—defined as minor cortical violations less than 2 mm—are widely considered clinically acceptable in the recent literature on cervical pedicle screws [4,11,14,15,16]. Only 5 screws (0.6% of 888 CPS) were classified as grade 3, indicating a major pedicle breach greater than 4mm. The rates of significant cortical breaches were low, and importantly none of these cases required revision surgery due to a misplacement.

Our findings are consistent with those of previous studies employing similar technologies and grading criteria. In our cohort, 94.5% of all cervical and thoracic screws were classified as grades 0 or 1. Gan et al. analyzed 297 navigated cervical pedicle screws and reported a 94.3% rate of radiographically satisfactory placement (grades 0 and 1), with no neurovascular injuries [4]. Similarly, Bredow et al. reported an accuracy (grade 0 and 1) of 78.5% for screws placed at C3–C7 and 93.9% at C1/C2 using 3D fluoroscopy-based navigation [11]. Ito et al. reported that 80% of screws were grade 0 and 20% grade 1, indicating high precision with navigation [17]. Richter et al. evaluated 31 cervical and 10 upper thoracic screws placed with navigation and found only one screw displaced by more than 1 mm, confirming reliable placement accuracy [18]. Scheufler et al., using a classification system comparable to Gertzbein–Robbins, reported that 99.3% of 248 screws were either grade 0 or 1, further supporting the high accuracy achievable with navigated techniques [19]. Similarly, Kumar et al. reported relatively low breach rates of 3.73% for grade 1 and 3.3% for grade 2 in a series of 241 cervical screws, noting that the highest breach rates tended to cluster at the C7 level [13].

Gan et al. also identified C5 as the level with the highest breach frequency (33.3%) [4]. In contrast, our cohort showed no clustering of high-grade breaches at any specific cervical level (see Table 1). Other authors, who investigated accuracy in robot-assisted surgeries, found rates of grades 0 and 1 between 94.5 and 98% [9,20,21].

Although the overall rate of grade 0 and grade 1 screw placements was high, grade 2 and grade 3 breaches were still observed in the cervical spine. In contrast, upper thoracic screw placement demonstrated notably high precision, with 92% of screws classified as grade 0 and the remaining 8% as grade 1. No grade 2 or 3 breaches occurred in the thoracic region, supporting the notion that the larger pedicle diameter and reduced segmental mobility in this area facilitate more accurate screw placement. Slight cortical violations (grade 1) were more frequently observed in the cervical spine (26.1%) compared to the thoracic levels (8.0%), which is also consistent with the existing literature suggesting that perfect screw placement in the cervical region is inherently more challenging [22]. The smaller pedicle diameter in the cervical spine increases the likelihood of minor cortical breaches, whereas the thoracic spine offers more favorable anatomy for precise instrumentation [14,23].

Moreover, due to the small pedicle size and implant-associated artifacts in intraoperative CT imaging, precise measurements at the millimeter scale can be difficult. Some screws may have been conservatively classified as grade 1 despite the absence of a true cortical breach, due to imaging artifacts or limited resolution that prevented a reliable differentiation between the pedicle cortex and the screw. However, as this was not systematically assessed, no specific size for this subset of screws can be provided. This phenomenon has also been described by Charles et al. in the context of thoracolumbar pedicle screw placement [24]. Richter et al. have pointed out that intraoperative imaging may underestimate true accuracy [18]. However, to avoid underestimating breach severity, all screws with uncertain grading in our study were consistently assigned to the worse Gertzbein–Robbins category. This mainly affected differentiation between grade 0 and grade 1 screws. Therefore, future studies should consider incorporating postoperative high-resolution CT scans alongside intraoperative cone beam-CT imaging to further investigate the reliability of accuracy measurements.

The overall revision rate in our cohort was 8.8%, with 3.4% attributed to screw loosening, 2.9% to rebleeding, and 2.0% to wound healing disorders. These rates are comparable to those reported in the literature [6]. For example, the incidence of SSI in cervical spine surgery has been reported at 3.4%, and 3.7% for thoracic spine surgery, particularly in posterior instrumentation procedures [25]. Our observed SSI rate of 2.0% therefore falls within the expected range for posterior cervical instrumentation.

Of clinical relevance is the absence of implant-associated neurovascular complications in our study. Even in four cases where intraoperative bleeding raised suspicion of vascular injury, subsequent angiographic imaging confirmed vessel integrity, and postoperative follow-up remained uneventful. These findings underscore the safety of the workflow and highlight the value of navigation in minimizing potentially catastrophic complications, which—although rare—remain a known risk in cervical spine instrumentation.

**Limitations:** This study has several important limitations. Its retrospective, single-center design limits generalizability and carries inherent risks of selection and reporting bias. Although this represents one of the largest series of navigated high-speed drill–assisted cervical pedicle screw (CPS) placements to date, the sample size may still be insufficient to detect very rare complications, such as vertebral artery injury.

Accuracy grading relied solely on intraoperative O-arm imaging, which, despite its utility, is prone to artifacts and limited resolution, and inter-rater reliability was not assessed. No comparator group (e.g., freehand or fluoroscopy-assisted techniques) was included, precluding direct evaluation of this workflow against other approaches. Furthermore, no formal statistical analyses or power calculations were performed, and subgroup analyses by surgeon experience, pathology, or construct length were not conducted.

While no screw-associated neural or vascular complications were observed, rare adverse events cannot be excluded, especially without routine postoperative angiographic follow-up in cases of suspected vascular injury. Finally, this study did not address cost implications, or outcomes in less specialized settings, and long-term patient-reported clinical outcomes were not assessed.

Future investigations should be carried out as prospective and multicenter studies, including postoperative high resolution CT images and control groups to confirm these findings and better define the risk profile of navigated CPS placement.

## 5. Conclusions

This study demonstrates that navigation-assisted cervical and upper thoracic pedicle screw placement using a high-speed drill can achieve high screw accuracy with a low observed complication rate. No revisions were required due to screw malposition, but 7 cases of screw loosening occurred. However, the retrospective design and reliance on intraoperative imaging limit the generalizability of the findings. Further prospective studies are needed to validate this workflow and assess its applicability in broader clinical settings.

## Figures and Tables

**Table 1:** Accuracy of cervical and upper thoracic pedicle screw placement by vertebral level and side. Values are shown as absolute numbers (n) and percentages (%). Percentages indicate the proportion of screws in each Gertzbein–Robbins grade relative to the total screws per segment and side. R = right side; L = left side; T = total (right and left side combined).

	Grade 0 (n)	Grade 1 (n)	Grade 2 (n)	Grade 3 (n)	Total	Grade 0 (%)	Grade 1 (%)	Grade 2 (%)	Grade 3 (%)
C2 R	56	22	10	0	88	63.6	25.0	11.4	0.0
C2 L	49	23	11	1	84	58.3	27.4	13.1	1.2
C2 T	105	45	21	1	172	61.0	26.2	12.2	0.6
C3 R	43	16	3	0	62	69.4	25.8	4.8	0.0
C3 L	45	14	2	1	62	72.6	22.6	3.2	1.6
C3 T	88	30	5	1	124	71.0	24.2	4.0	0.8
C4 R	51	20	2	0	73	69.9	27.4	2.7	0.0
C4 L	46	18	5	0	69	66.7	26.1	7.2	0.0
C4 T	97	38	7	0	142	68.3	26.8	4.9	0.0
C5 R	48	20	8	0	76	63.2	26.3	10.5	0.0
C5 L	44	25	4	1	74	59.5	33.8	5.4	1.4
C5 T	92	45	12	1	150	61.3	30.0	8.0	0.7
C6 R	58	17	4	1	80	72.5	21.2	5.0	1.2
C6 L	54	24	2	0	80	67.5	30.0	2.5	0.0
C6 T	112	41	6	1	160	70.0	25.6	3.8	0.6
C7 R	53	12	4	1	70	75.7	17.1	5.7	1.4
C7 L	48	21	1	0	70	68.6	30.0	1.4	0.0
C7 T	101	33	5	1	140	72.1	23.6	3.6	0.7
T1 R	61	5	0	0	66	92.4	7.6	0.0	0.0
T1 L	60	6	0	0	66	90.9	9.1	0.0	0.0
T1 T	121	11	0	0	132	91.7	8.3	0.0	0.0
T2 R	31	5	0	0	36	86.1	13.9	0.0	0.0
T2 L	36	0	0	0	36	100.0	0.0	0.0	0.0
T2 T	67	5	0	0	72	93.1	6.9	0.0	0.0
T3 R	8	2	0	0	10	80.0	20.0	0.0	0.0
T3 L	10	0	0	0	10	100.0	0.0	0.0	0.0
T3 T	18	2	0	0	20	90.0	10.0	0.0	0.0
Total	801	250	56	5	1112	72.0	22.5	5.0	0.4
Cervical	595	232	56	5	888	67.0%	26.1%	6.3%	0.6%
Thoracic	206	18	0	0	224	92.0%	8.0%	0.0	0.0

**Table 2:** Causes and frequency of revision surgeries within the cohort.

Reason for Revision	n	% (of Cohort)	Time Interval * (Days)	Mean Time Interval (Days)	Note
Screw loosening	7	3.4%	28, 61, 97, 135, 152, 163, 233, 302	130.1	In 2 cases, not CPS but lateral mass screw failure led to revision
Rebleeding	6	2.9	0, 0, 0, 1, 1, 3	0.8	
Wound healing disorder	4	2.0	11, 28, 21 and 35	23.8	
Total	18	8.8	**-**	**-**	

* Time interval between index surgery and revision surgery.in days for each patient in whom revision surgery was necessary, e.g., 0 means occurrence of the complication on same day as index surgery, 2 on the second day after index surgery.

## Data Availability

The data that support the findings of this study are available from the corresponding author, upon reasonable request.

## Abbreviations

The following abbreviations are used in this manuscript:

CPS	Cervical pedicle screw
CT	Computed tomography
